# Feasibility of the Go2Play Active Play intervention for increasing physical and social development in children with intellectual disabilities

**DOI:** 10.1186/s40814-021-00783-6

**Published:** 2021-02-05

**Authors:** Arlene McGarty, Nathalie Jones, Katie Rutherford, Sophie Westrop, Lara Sutherland, Andrew Jahoda, Craig Melville

**Affiliations:** Institute of Health & Wellbeing, University of Glasgow, 1st floor Admin Building, Gartnavel Royal Hospital, 1055 Great Western Road, Glasgow, G12 0XH Scotland, UK

**Keywords:** Physical activity, Children, Intellectual disabilities, Active play, Fundamental movement skills, Social skills

## Abstract

**Introduction:**

An active play is designed to increase children’s physical activity levels and fundamental movement skills through outdoor play and is well-suited to the needs of children with intellectual disabilities. However, no active play interventions have included children with intellectual disabilities. This study aims to investigate the feasibility of a school-based active play intervention for children with intellectual disabilities.

**Method:**

Children aged 7–12 years who had intellectual disabilities and were independently ambulatory were eligible. This single-group 17-week intervention was implemented in two additional support needs schools. It consisted of a weekly 1-h active play session incorporating 30 min of structured games and 30 min of free play. Feasibility of recruitment/retention, adherence, and outcome measures were investigated. Outcome measures included school-based physical activity (ActiGraph GT3X+ accelerometer), fundamental movement skills (Test of Gross Motor Development-2), and social interactions (Playground Observation of Peer Engagement). Staff feedback was collected via open-ended questionnaire. Feasibility was investigated using descriptive statistics and questionnaire data analyzed using thematic analysis. Potential pre-post changes were investigated for school-based physical activity, fundamental movement skills, and social interactions using paired samples *t* tests. The progression criteria were (1) > 50% of eligible participants recruited, (2) > 50% of recruited participants retained, (3) > 50% of active play sessions spent in MVPA, and (4) > 50% of participants complete outcome measurements.

**Results:**

All progression criteria were met. Recruitment and retention rates were 100% (*n*=21 participants). Intervention adherence was high, based on data from *n*=1 school, with 90% of participants attending all sessions. Measuring physical activity using accelerometry and fundamental movement skills using the Test of Gross Motor Development-2 were feasible. The Playground Observation of Peer Engagement tool to measure social interactions was not feasible. The only significant increase post-intervention was for social interactions during structured play (pre–post mean difference: –1.46, 95% CI −1.99, −0.93). Staff feedback was positive with the intervention well received by schools and potential benefits post-intervention identified by teachers.

**Conclusion:**

The Go2Play Active Play intervention is feasible for children with intellectual disabilities. Future research should further investigate feasibility and implementation on a larger scale using a pilot cluster randomised controlled trial.

**Trial registration:**

ISRCTN registry: ISRCTN10277566.

## Introduction

Physical activity refers to any bodily movement produced by the skeletal muscles that increases energy expenditure above a resting level [1] and is associated with numerous physical and mental health benefits in children, e.g. reduced levels of obesity, anxiety, and depression [2–4]. It is recommended that children participate in an average of 60 min moderate to vigorous physical activity (MVPA) per day and minimise sedentary time [5]. However, many children with intellectual disabilities, which refers to limitations with intellectual functioning (intelligent quotient: IQ < 70) and adaptive behaviours [6], complete insufficient activity to gain clinically meaningful health benefits and spend a large portion of their day sedentary [7, 8].

As childhood physical activity levels are predictive of activity in adulthood, it is essential to develop methods to increase childhood activity to promote long-term activity and health [9]. However, no interventions have been effective at increasing physical activity levels in children with intellectual disabilities [10]. Previous interventions have generally focussed on exercise training rather than being tailored to overcome known barriers to activity [10]. Children with intellectual disabilities can face various barriers to physical activity, including not having the physical skills required to be active, reduced social skills, and a lack of accessible clubs and facilities [11]. These intrapersonal and environmental barriers limit the opportunities that children with intellectual disabilities have for physical activity, which needs to be addressed.

It is therefore important to develop new methods that address structural barriers to activity that are not only focussed on individual behaviour change. Interventions should focus on long-term physical activity, address known barriers to activity, and support children with intellectual disabilities to develop the skills and motivation required to be active throughout their lives [10]. One evolving method warranting further investigation is ‘active play’, which is ‘a form of gross motor or total body movement in which young children exert energy in a freely chosen, fun, and unstructured manner’ (p.164) [12]. Active play is mostly conducted outdoors and therefore is not restricted to one location, is suitable for varying levels of abilities, and requires little or no equipment [12]. Outdoor time is associated with increased physical activity and cardiorespiratory fitness and reduced sedentary time [13]. The evidence based on the benefits of active play is still limited, but the emerging findings suggest it has the potential to increase children’s MVPA levels and fundamental movement skills [14]. Therefore, as active play requires minimal infrastructure and is designed to develop the fundamental physical and social skills required to be active, this could help children with intellectual disabilities to overcome the barriers to activity that they commonly face.

Recently within the UK, the Go2Play Active Play intervention has been implemented in mainstream primary schools as part of a pragmatic evaluation and a feasibility cluster randomised controlled trial [15, 16]. This intervention was shown to be feasible to implement in schools, effective at getting children active at a moderate-vigorous intensity, and provided preliminary evidence of positive intervention effects, e.g. increased MVPA and fundamental movement skills. However, these studies did not include additional support needs schools, and therefore, no data is available on the feasibility of this intervention for children with intellectual disabilities. As the ‘key ingredients’ of behaviour chance within this intervention relate to children developing the basic physical and social skills that are required to be active, it is well suited to the needs of children with intellectual disabilities.

The overall aim of this study is to investigate the feasibility of the Go2Play Active Play intervention for children with intellectual disabilities in additional support needs schools. This will enable data to be gathered on the feasibility key outcomes that will inform the development of a larger trial. This study was designed based on the Medical Research Council guidelines for developing and evaluating complex interventions, specifically the phase of testing trial feasibility/piloting [17]. Therefore, this feasibility study aims to investigate (1) recruitment and retention, (2) intervention adherence, (3) the feasibility and acceptability of different outcome measures, and (4) potential intervention effects.

## Methods

### Ethical approval

Ethical approval for this study was granted by the Medical, Veterinary, and Life Sciences College ethics committee, University of Glasgow.

### Design

A single-group design was employed. The intervention was delivered in two additional support needs schools in Greater Glasgow, Scotland, during one school term (January to June, 2019). Baseline measurements were conducted in the week prior to the intervention commencing and post-intervention measurements were conducted the week after the intervention ended. This trial was registered with the ISRCTN registry (ISRCTN10277566).

### Recruitment and participants

A convenience sample was recruited from two additional support needs schools. Two classes were selected from each school to recruit from (*n* = 22 children in total), with the aim of recruiting all of these children. This sample size was chosen as it is sufficient for the initial feasibility aims to be assessed. Only recruiting from specific classes was a feasibility decision as it enabled the Active Play sessions to be conducted at a class level. A structured recruitment strategy was utilised, with eligible children given an information pack with study details to discuss with their parents. This was followed-up with a parent information session at each school, at which parents could ask questions and get more information on the study. All aspects of recruitment were conducted by one researcher who was not involved in any other aspects of data collection. Children were eligible to participate if they had intellectual disabilities, were aged 7–12 years, and were independently ambulatory. Intellectual disability was measured using the Child and Adolescent Intellectual Disability Screening Scale (CAIDS-Q), which enables a yes/no assessment on the likelihood of intellectual disabilities based on age-specific criteria. Children with additional support needs who did not have intellectual disabilities were not eligible. All participants and parents were required to provide informed consent.

### Go2Play Active Play intervention

The Go2Play Active Play intervention was developed in partnership between Agile CIC social enterprise (www.agilecic.com) and Inspiring Scotland (www.inspiringscotland.org.uk) to encourage children to play physically active games in an outdoor environment. It is underpinned by the concept of physical literacy and the development of the fundamental movement skills, motivation, and confidence required to be physically active. In addition to skill development, this intervention primarily aims to support behaviour change in children through increased social support and environmental change [18]. Prior to the intervention commencing, one of the intervention development team observed physical education classes at the participating schools to assess if any adaptations were required to the intervention. Due to the flexibility of the programme, no adaptations to content of the active play sessions were deemed necessary. However, due to developmental differences between children with and without intellectual disabilities, it was deemed appropriate to recruit an older sample of children (7–12 years) in comparison to the previous studies in mainstream schools, which recruited 7-year-old children [15, 16]. Furthermore, in mainstream schools the intervention was delivered for 10 weeks with two sessions per week. However, due to the demands of the curriculum in the participating schools it was not possible to deliver sessions within this timeframe. Instead, the intervention was delivered once per week over a longer duration. Otherwise, the intervention remained the same as the version delivered in the mainstream schools [15, 16].

The intervention ran for 17 weeks (excluding school holidays) and consisted of a weekly 60-min active play session. Sessions were facilitated by the same local play charities who delivered the Go2Play Active Play intervention in mainstream schools. Training about children with intellectual disabilities was given to the play workers who were delivering the intervention, e.g. how to communicate instructions. The sessions were designed to develop fundamental movement skills and increase activity levels, but also to be fun and inclusive to foster positive experiences and perceptions of physical activity. Sessions consisted of a 30-min structured play, with sessions focussed on developing different fundamental movement skills, e.g. throwing and catching games to develop object control. The second half of the session was free play where children were encouraged to interact and to create and play their own games, with the aim of developing social skills.

### Demographic and developmental variables

Anthropometric measurements were taken at baseline and post-intervention, in accordance with the International Standards for Anthropometric Assessment [19]. Weight and height were measured using digital scales and a stadiometer, respectively, and body mass index calculated [BMI: weight/height^2^ (kg/m^2^)]. Parents of participants also reported their child’s age and gender via questionnaire. The British Picture Vocabulary Scale (BPVS) [20] was used to assess the language skills of participants. Teachers completed the seven-item CAIDS-Q, which strongly correlates with IQ and adaptive behaviour scores, and accurately determines whether children have intellectual disabilities [21]. The CAIDS-Q was used to ensure that all participants had an intellectual disability.

### Feasibility and acceptability (primary outcomes)

#### Recruitment and retention

Recruitment rates were calculated as a percentage based on the number of children recruited vs. the number of children in the four participating classes (*n* = 22). Data relating to recruitment was collected by a member of the research team not directly involved in delivering the intervention or conducting any pre-post measurements. Dropout was defined as any child (at their own or at their parent’s request) who chose to no longer participate in the active play sessions and/or did not to participate in any of the baseline or post-intervention measurements; teachers and play workers were asked to inform the research team if any participants dropped out of the study. These outcomes are consistent with those used when this intervention was delivered in mainstream school, which enables direct comparison.

#### Intervention adherence

Adherence was operationalised as attendance at the active play sessions, with all non-attendance recorded as non-adherence, and session MVPA levels. Teachers were asked to record participant attendance at the active play sessions and reasons for non-attendance. The sessions were designed with the aim of children completing at least 30 min (50% of each session) in MVPA. Participants wore an ActiGraph wGT3X+ accelerometer during four sessions to assess whether at least 50% of the session in was spent doing MVPA. The ActiGraph wGT3X+ is a small, lightweight device that been validated for children with intellectual disabilities and was worn around the waist, attached using an elastic belt [22]. Physical activity was recorded using 5-s epochs, and due to the active nature of the sessions, 5 min of continuous zero counts was used to identify non-wear. MVPA was classified using population-specific cut points [22].

#### School-based physical activity and sedentary behaviour

School-based physical activity and sedentary behaviour were measured using the ActiGraph wGT3X+ accelerometer. Participants wore the accelerometer during school hours for five consecutive days at baseline and post-intervention. We also asked teachers to complete an accelerometer wear diary to record when the devices were put on and taken off for each participant, and to report reasons for the device being removed during the day. The required wear time for the device was four out of the five measurement days for at least 3 h per day (which represents approximately 50% of the school day), and non-wear was classified as 20 min of continues zero counts [23, 24]. We recorded how many participants met this criteria to assess the feasibility and acceptability of this measure. Sedentary behaviour and physical activity levels were estimated from the raw count data using population-specific cut points [22].

#### Fundamental movement skills

Fundamental movement skills were assessed at baseline and post-intervention using the Test of Gross Motor Development-2 (TGMD-2), which is valid and reliable for children with intellectual disabilities [25]. This test measured locomotor skills (e.g. running and jumping) and object control (e.g. kick and throw), the results of which give an overall gross motor quotient. The percentage of participants completing this test, as well as reasons for non-compliance, was recorded to assess feasibility. Participants removed their accelerometers during these measurements.

#### Social interactions

Social interactions were measured during the first and last intervention sessions using the Playground Observation of Peer Engagement (POPE) tool [26]. This time-interval behavioural coding tool is designed to assess the social interactions of children with additional support needs during play. This was conducted using direct observation, with the sessions video-recorded and analysed post hoc by one researcher. Behaviours were coded by observing for 40 s and coding behaviours for 20 s for each minute of the session. The RESAM component of this scale was used to give an overall score based on five types of social behaviours during play: (1) initiated interaction with another child, (2) responded to another child, (3) engaged in a conversation (≥ 4 exchanges with another child), (4) engaged in a game with another child/group of children, and (5) observed a game of another child/group of children. Participants were scored when they demonstrated one of these behaviours and the final rating ranged from 0 (demonstrated none of these behaviours) to 5 (demonstrated all these behaviours). Feasibility was assessed on whether this direct observation methodology and use of the POPE tool enabled all relevant social interactions to be recorded.

#### Staff feedback

A questionnaire was developed to collect qualitative data from teachers (*n* = 3), whose students were involved in the intervention, and the lead facilitator of the intervention (*n* = 1). The questionnaire contained seven open-ended questions relating to perceptions of feasibility, benefits/limitations, and effectiveness of delivery. Questionnaires were completed in paper (*n* = 3) or electronic (*n* =1) format.

### Potential intervention effects (secondary outcomes)

Potential intervention effects were examined using baseline and post-intervention data for MVPA, sedentary behaviour, social interactions, and fundamental movement skills.

### Statistical analysis

Quantitative primary outcome and demographic variables were analysed descriptively, with recruitment, retention, and session adherence rates calculated as percentages. Qualitative data were analysed by one researcher using thematic analysis in accordance with Braun and Clarke’s six-phase approach [27]. Although this study was not powered to detect change, potential pre-post changes were investigated for school-based MVPA and sedentary behaviour, fundamental movement skills, and social interactions using a per protocol analysis with paired samples *t* tests. All statistical data were analysed using SPSS 21 statistical package (SPSS IBM, New York, NY, USA), and results are presented as mean (M) and standard deviation (SD) unless otherwise stated. Statistical significance was set at *p*<.05. The criteria to assess whether this study should progress to a larger trial were (1) > 50% of eligible participants recruited, (2) > 50% of recruited participants retained throughout the trial, (3) > 50% of active play sessions spent in MVPA, and (4) > 50% of participants complete outcome measurements.

## Results

### Participant summary/demographics

Twenty-two participants took part in this study. However, one participant’s CAIDS-Q score was not in the intellectual disabilities range and their data were excluded from the analyses, resulting in a final sample of *n*=21. Participants were aged from 7 to 12 years, and most were male and ranged from underweight to obese. The participants’ demographic data are presented in Table 1.

**Table 1:** Demographic data of participants

Demographic variable	
Age (years)	*M* = 9.57 (SD = 1.21)
Sex	
Male	*n* = 16 (76.20%)
Female	*n* = 5 (23.80%)
Height (m) (*n* = 20)	*M* = 1.43 (SD = 0.11)
Weight (Kg) (*n* = 20)	*M* = 42.00 (SD = 14.38)
BMI (kg/m^2^) (*n* = 20)	*M* = 20.07 (SD = 4.58)
Underweight	*n* = 2 (9.5%)
Healthy weight	*n* = 10 (47.6%)
Overweight	*n* = 2 (9.5%)
Obese	*n* = 6 (28.6%)
BPVS raw scores (*n*=19)	*M* = 61.89 (SD = 28.50)
CAIDS-Q score (%) (*n*=20)	*M* = 31.55 (SD = 12.51)

Note: Data presented from *n*=21 participants unless stated otherwise

*BMI* body mass index, *BPVS* The British Picture Vocabulary Scale, *CAIDS-Q* Child and Adolescent Intellectual Disability Screening Scale, *M* mean, *SD* standard deviation

### Primary outcomes: feasibility and acceptability of measures

#### Recruitment and retention

Across the two schools, we had a 100% recruitment rate. All children (*n*=21) who were eligible to take part provided the required child and parent consent, demonstrating that the recruitment strategy was feasible and effective. In relation to acceptability, we had a 100% retention rate with no participants withdrawing. Results are summarised in Table 2.

**Table 2:** Summary of the feasibility results

Feasibility outcome	Result
Recruitment and retention strategy	Feasible
Intervention adherence	Feasible
School-based accelerometery measurement	Feasible
Test of Gross Motor Development-2 (TGMD-2)	Feasible
Playground Observation of Peer Engagement (POPE) tool	Not feasible^a^

^a^POPE tool was not feasible but the direct observation methodology was feasible

#### Intervention adherence

Only one school provided data relating to intervention adherence, which reported 90% of participants attended all sessions for the full duration. Reasons for non-attendance were mostly due to participants being absent from school but behavioural reasons were also reported. Based on accelerometer data collected during the four sessions, participants spent *M* = 57.11 % (SD = 20.78%) of the sessions in MVPA. Although sessions were intended to be 60 min, the average session duration observed over the four sample sessions was *M* = 40.60 min (range: 39.00–41.12 min) due to the time required to get participants ready and to manage any challenging circumstances. The split between structured and free play was consistent with the intended 50/50 split, with the average split across the four sample sessions being *M* = 21.50 min structured play and *M* = 18.5 min free play.

#### Feasibility of outcome measures

For school-based physical activity and sedentary behaviour, *n*=12 (57.14%) met the accelerometer wear criteria (4 out of 5 days for ≥ 3 h per day) at baseline and *n*=13 (61.90%) met the criteria at post-intervention. However, 8 accelerometers malfunctioned at baseline due to an error that occurred during group initialisation and no data was collected. Therefore, assessing wear time for these participants, who all attended the same school, was based on data collected from the wear diaries. Reasons for non-compliance with the wear criteria were primarily due to absences from school, with *n*=2 participants refusing to wear the accelerometer at baseline and post-intervention.

For fundamental movement skills, *n*=20 (95.24%) participants completed the measurements at baseline, with one participant refusing. Post-intervention, *n*=19 (90.48%) participants completed the measurements, with two participants absent. In relation to feasibility, minor issues were encountered with finding an available space within the schools that was a suitable sized and quiet enough to conduct the tests. In addition, for some parts of the tests, participants were assisted by teachers, e.g. hand holding.

For the social interactions measurements, baseline and post-intervention data were collected for *n*=17 (80.95%) and *n*=12 (57.14%) participants, respectively, with all missing data due to absences from school. However, during the analysis issues were identified with the feasibility of the POPE tool as it is designed to only measure children’s social behaviour, independent of adult assistance. During the sessions, however, it was observed that adult presence and assistance (e.g. play workers and teachers) was unavoidable due to the design of the intervention and the additional support needs of the children. Therefore, the analysis included social interactions that also involved adults, which limits the validity and applicability of this tool.

#### Staff feedback

Themes identified from the questionnaires were practicalities and scheduling, engagement with the intervention, and behaviour changes over the intervention duration. In summary, feedback was highly positive from teachers and the play worker who all noted high levels of enjoyment and engagement from participants during the sessions. Teachers reported positive benefits for participants as the intervention progressed, such as increased class-time concentration, willingness to participate, and improved physical and social skills. The play worker also reported that the original intervention design that was implemented in mainstream schools was feasible and did not require significant changes. The challenges discussed included finding time to schedule the sessions towards the end of term and the practical difficulties of conducting baseline and post-interventions in the weeks close to major school holidays, as the number of extra-curricular activities increased during these times. It was also noted that occasionally some participants could not participate in all activities without extra staff attention.

### Secondary outcomes: potential intervention effects

#### School-based physical activity and sedentary behaviour

Accelerometer data from the 5-day pre- and post-intervention weeks showed similar time spent in MVPA and sedentary behaviour. MVPA (percentage of wear time) was *M* = 16.87% (SD = 11.84) at baseline and *M* = 15.45 % (SD = 8.03) post-intervention, which shows no indication of change (pre–post mean difference: 1.42, 95% CI − 3.62, 6.47). Sedentary behaviour (percentage of wear time) was *M* = 71.05 % (SD = 13.83) at baseline and *M* = 73.00 % (SD = 11.72) post-intervention, which also shows no indication of change (pre–post mean difference: –1.95, 95% CI − 7.29, 3.39).

#### Fundamental movement skills

Gross motor quotient scores were *M* =59.50 (SD = 12.46) at baseline and *M* = 62.60 (SD = 14.11) post-intervention, which shows no indication of change (pre–post mean difference: –3.40, 95% CI − 7.46, 0.66).

#### Social interactions

For the structured part of the sessions, out of a possible score of 5, scores were *M* = 2.77 (SD = 1.26) at baseline and *M* = 4.23 (SD = 1.42) post-intervention, which represents a significant increase in social interactions (pre–post mean difference: –1.46, 95% CI − 1.99, − 0.93). During the free-play component of the sessions, there was an increase in scores from baseline (*M* = 3.45, SD = 1.51) to post-intervention (*M* = 4.00, SD = 1.27), but this was not significant (pre–post mean difference: –0.55, 95% CI − 1.93, 0.84).

## Discussion

This study is the first to examine the feasibility of delivering a school-based active play intervention to children with intellectual disabilities. The results of this study suggest that the Go2Play Active Play intervention is feasible for children with intellectual disabilities.

We had excellent recruitment and retention rates (100%) for the study. This is equal or higher than the rates reported when this intervention was implemented in mainstream schools, which reported recruitment and retention rates of 66% and 100% [15, 16]. However, it is important to note that the participating schools had existing relationships with the research team, which may have increased school/teacher engagement. Nonetheless, this is a positive finding and highlights that it is feasible to recruit and retain children with intellectual disabilities to an active play intervention. The difficulties with recruiting children with intellectual disabilities to research are well reported [28]. Therefore, this demonstrates that the recruitment strategy is effective.

Adherence with the intervention was also high, with 90% of participants attending all sessions. None of the reasons for non-attendance were related to the study and were due to school absences or behavioural issues. In the context of additional needs schools, it is common for children to be absent for part of the school day to attend appointments, e.g. hospital appointments or therapy session, and for children to occasionally display behavioural problems [29]. Therefore, it may be difficult to overcome these issues in future studies. Only one school reported adherence rates, however, due to teacher workload and as such this rate may not be fully representative and therefore should be interpreted with caution. To address this, in future phases of this work, adherence rates should be recorded by members of the research team or play workers, rather than teachers. Another important point to note is that the age of participants in this study ranged from 7–12 years. As demonstrated by the high levels of adherence and recruitment, this highlights that the flexibility of the intervention enables it to be suitable for a wide range of participants, which is a promising finding in relation to scaling up this intervention.

Participants also spent a large portion of the sessions active, demonstrating the effectiveness of the intervention to get participants active at a moderate to vigorous intensity. Therefore, the intervention design does not need to be changed for future phases. This demonstrates that this intervention could contribute to children with intellectual disabilities achieving the physical activity guidelines and address the low levels of school-based physical activity and, specifically, low levels of MVPA [30, 31]. However, this study did highlight that although the sessions were designed to last 60 min, the mean session time was 40.60 min, which was a consistent session duration across the two schools (range: 39.00–41.12 min). This was due to the time taken to get participants from classes and changed before and after the session. This is not an issue specific to additional support needs schools as this was also reported when the Go2Play Active Play intervention was implemented in mainstream schools [16]. However, to ensure consistency of delivery in future phases of this work, it is important to account for this and work with schools to try and ensure the session duration is 60 min.

Based on teacher feedback, future phases of this work should also consider when baseline and post-intervention measurements are conducted. The baseline measurements were conducted the first school week after the Christmas break and the post-intervention measurements were conducted during the final week before the summer holidays. Due to these measurements being close to major school holidays, there were numerous children who missed parts of these measurements, in particular the post-intervention measurements, which could impact the statistical power of a future study. Therefore, when to conduct baseline and post-intervention measurements should be discussed with the schools to reduce this effect.

In relation to the outcome measures, it is a promising finding that all measures were feasible, with the exception of the POPE observation tool. Although it is important to note that direct observation was feasible, even though the recording categories of the POPE tool proved to be unsuitable. Compliance with wearing the accelerometer was high when children were in school. However, the high level of absences during the measurement weeks prevented some participants from achieving the wear criteria. If the data collection weeks can be arranged in consultation with schools, then it may be possible to improve compliance. If not, the wear criteria may have to be reduced to accommodate the unique environment of additional support needs schools. Finally, the role that adult support had on measurements and how this impacted the validity of test results needs to be considered. This was particularly apparent for the TGMD-2, and therefore, future studies should limit teacher involvement during the TGMD-2 by including additional researchers to support the administration of this test.

Teacher and play worker feedback provided valuable data on how the intervention was received within the school and by the participating children. It was highlighted that the start and end of the school term are busy and therefore not the most effective times to conduct baseline and post-intervention measurements. These views were consistent with the participant data. Another interesting outcome from this qualitative element was the benefits reported by teachers as the intervention progressed, such as improved social skills during class time. This is an interesting finding and supports the pre-post analysis which found significant increases in social interactions post-intervention. Therefore, it is important to investigate these outcomes in future phases of this work with a sample size that is sufficiently powered to detect change. One of the challenges reported by the play workers was that some children could not participate in all aspects of the session without adult support. This was to be expected due to the wide-ranging intellectual disabilities of participants, which was why adult support was encouraged, where necessary, during the sessions. As the original Go2Play Active Play intervention was designed for children aged approximately 7 years, this also suggests that this intervention is better suited to older children with intellectual disabilities due to differences between their chronological and developmental age. However, the suitability of this intervention to children with intellectual disabilities of different ages requires further examination. In addition, it is also important to conduct a more in-depth process evaluation to better capture data on the implementation of this intervention.

### Future research needs

In line with the Medical Research Council guidelines for developing and evaluating complex interventions [17], future research should focus on piloting the Go2Play Active Play intervention in a larger and representative sample of children with intellectual disabilities, with a wider range of ages included. A pilot cluster randomised controlled trial with a wait-list control group would enable preliminary assessment of effectiveness, e.g. increasing physical activity levels, fundamental movement skills, and social skills, estimating sample size for a full trial, and test the feasibility of randomising schools.

### Strengths and limitations

This study addresses a significant gap in the literature and proposes a novel approach for increasing physical activity in children with intellectual disabilities. A thorough investigation of feasibility and acceptability was conducted using both quantitative and qualitative methods, which has enabled in-depth data to be collected that can inform future phases of this work. Limitations are that the participating schools were selected based on existing relationships with the research team, which may have increased buy-in and engagement from the head teacher and teaching staff. In addition, participants were recruited from selected classes, and therefore, the findings from this sample may not be fully representative of all children attending the schools; this is further highlighted by 76.2% of the sample being male.

## Conclusions

In conclusion, this study has demonstrated that it is feasible to implement the Go2Play Active Play intervention in schools for children with additional support needs. The design and measures are feasible and acceptable, although some aspects of data collected may need to be modified for a future trial. The intervention received positive feedback from staff, and potentially positive changes were identified from both the qualitative and quantitate elements of this study. For the next phase of this study, it is essential to test the effectiveness of this intervention in a greater number of schools, with sufficient statistical power to detect pre-post changes, and to conduct a more in-depth process evaluation to further investigate implementation, e.g. and feasibility on a larger scale.

## Data Availability

The datasets generated and/or analysed during the current study are not publicly available due to restrictions with the ethical approval but are available from the corresponding author on reasonable request.

## Abbreviations

BMI: Body mass index
BPVS: British Picture Vocabulary Scale
CAIDS-Q: Child and Adolescent Intellectual Disability Screening Scale
IQ: Intelligent quotient
*M*: Mean
MVPA: Moderate to vigorous physical activity
POPE: Playground Observation of Peer Engagement
SD: Standard deviation
TGMD-2: Test of Gross Motor Development-2
